# Using attractiveness model for actors ranking in social media networks

**DOI:** 10.1186/s40649-017-0040-8

**Published:** 2017-06-26

**Authors:** Ziyaad Qasem, Marc Jansen, Tobias Hecking, H. Ulrich Hoppe

**Affiliations:** 1 Computer Science Institute, University of Applied Science Ruhr West, Lützowstraße 5, 46236 Bottrop, Germany; 2 Dept. of Computer Science and Applied Cognitive Science, University of Duisburg-Essen, Lotharstraüe 63, 47057 Duisburg, Germany

**Keywords:** Actor influence, Social media networks, Twitter, IC model, Information diffusion, Independent cascade model, T measure

## Abstract

**Background:**

Influential actors detection in social media such as Twitter or Facebook can play a major role in gathering opinions on particular topics, improving the marketing efficiency, predicting the trends, etc.

**Proposed methods:**

This work aims to extend our formally defined *T* measure to present a new measure aiming to recognize the actor’s influence by the strength of attracting new important actors into a networked community. Therefore, we propose a model of the actor’s influence based on the attractiveness of the actor in relation to the number of other attractors with whom he/she has established connections over time.

**Results and conclusions:**

Using an empirically collected social network for the underlying graph, we have applied the above-mentioned measure of influence in order to determine optimal seeds in a simulation of influence maximization. We study our extended measure in the context of information diffusion because this measure is based on a model of actors who attract others to be active members in a community. This corresponds to the idea of the IC simulation model which is used to identify the most important spreaders in a set of actors.

## Background

With the wide spread of social media networks nowadays, it has become possible to acquire insights into and knowledge about a wide variety of more or less numerous communities interacting through the Internet. Moreover, applying analytic approaches to social media data can provide better-informed decision-making processes in various fields such as marketing, politics, and education. In fact, there is an important aspect of such analytics, that is, the detection and characterization of influential actors in social networks. Various studies have suggested different approaches and specific measures to solve the problem of influential actors detection.

Influential actors in social media have an effective role in information diffusion. For instance, a viral marketing operation for a new product can be conducted by seeding the product in Twitter with a few elected influential actors who can influence others in a way that might help in the rapid spread of that product.

*T* measure [2, 3] provides a new type of influence in online social network in order to emphasize those actors who attract many outsiders to join the own community in which a specific topic is dealt. For example, in Twitter, those actors spawn many retweets on a certain topic from people who have no previous contributions on that topic.

In this paper, we elaborate on a new extended measure *HT* for the detection of influential actors, which is based on quantifying the contribution of this actor to increasing the size of the network by attracting new active members of the specific subcommunity [4]. In other words, while *T* measure defines the attractiveness value of an actor through evaluating the number of outsiders who joined to the community by this actor, *HT* measure will refer to his/her attractiveness value through evaluating the importance of those outsiders. In the evaluation section of this paper, we apply our approach first to dataset from the Asterisk open source software developer community (a relatively small community with less than 1400 members and much less active actors). As well as, we apply the measure to a dataset based on Twitter communication around #EndTaizSiege (related to recent events in Yemen). We study the relation between our measure and other influence measures by computing the correlation values between them. Furthermore, we compare our measure with *T*, Katz centrality, PageRank, indegree, and betweeness measures in terms of how good these measures are if used to refer to the influential actors in social media in terms to their ability to attract others to become active in the information diffusion process.

The rest of the paper is organized as follows: “Literature review” section presents related research. Basic formal definitions of our approach are given in “Approach” section, which also provides the implementation of *T* and *HT* measures. “Evaluation I” section describes our datasets and the experimental results. “Evaluation II” section deals with the performance of our measure in the influence maximization problem. Finally, conclusions are drawn and an outlook for further research is described in “Conclusions” section.

## Literature review

Social influence analysis has attracted considerable research interests in recent years. A wide scheme of research focused on modeling and measuring influence and on influential actors detection. Particularly, online social networks such as Twitter are of special interest. However, regarding the manifestation and identification, there are still open questions.

It could be shown from the study presented by Cha et al. [5] that applying different measures can produce utterly different results when it comes to the task of ranking actors according to their influence. They illustrated an in-depth comparison of three measures of influence: indegree (number of followers of an actor), retweets (number of retweets containing one’s actor name), and mentions (number of mentions containing one’s actor name). They concluded that different measures can be used to identify different types of influential actors. Popular actors with high indegree were not necessarily influential in terms of spawning retweets or mentions and most influential actors can hold significant influence over a variety of topics. Consequently, the way in which a network is extracted from social media content and the measure of influence should be considered carefully with respect to the roles and type of influence one aims to reveal.

Azaza et al. [6, 7] proposed a new influence assessment approach depending on belief theory to combine different types of influence markers on Twitter such as retweets, mentions, and replies. They used Twitter dataset of European Election 2014 and deduced the top influential candidates.

Qasem et al. [2, 3] proposed a new approach which is related to the research presented in [5] in the sense that it aimed for a clear formulation of social influence and a methodology to produce an exact ranking of the actors according to the definition. In concrete, Qasem et al. [2] introduced a new type of influence in online social network to define those actors who attract many actors to join the own community in which a specific topic is dealt. Based on this type of influence, a new measure (*T* measure) has been proposed to define those actors.

In contrast to local measures that only take into account the direct neighborhood of an actor, there exist also recursive measures that determine the centrality of an actor relative to the influence of its neighbors. A measure of influence proposed in the early years of social network analysis, which is still of importance, is the Katz centrality [8]. It accounts for the ability of an actor to spread information through a network by counting the number of paths the actors have to each other actor. In addition, longer paths are weighted less than short paths.

Closely related measures are Eigenvector centrality for undirected networks and PageRank for directed networks [9]. These measures are recursive in the sense that they calculate the centrality of each actor based on the centrality of its neighbors. These ideas were taken up in this work to assess the importance of an actor according to the potential to attract new actors to join the network. Here, the attraction value of an actor can be adjusted by the attraction values that the attracted actors achieve later on. In other words, high attractors are those who influence others to become active in the Twitter communication and attract many others to do so.

Information diffusion in a network refers often to the influence in the spread of information. Particularly in social media, influential actors can control the diffusion of information through the network to some extent. Information diffusion is defined as the process by which a new knowledge or idea spread over the social networks by the means of communications among the social network actors [10]. The most widely used information diffusion models are the independent cascade (IC) [11, 12] and the linear threshold (LT) [13]. These two models describe different aspects of influence diffusion. The IC and LT models have been introduced by Kempe et al. [14] to fix the problem of the influence maximization which search for those actors whose aggregated influence in the social network is maximized. However, Pei et al. [15] provided strategies to search for spreaders based on the following of information flow rather than simulating the spreading dynamics (modeled_dependent results). The research of [14] was followed by many studies which discuss the same problem (e.g., [16–18]). Furthermore, The features of identifying spreaders measures using independent interaction and threshold models through empirical diffusion data from LiveJournal are discussed in [19]. Morone et al. [20] proposed to map the problem of influence maximization in complex networks onto optimal percolation using CI (collective influence) algorithm.

Our work is related to the research presented in [2] in the sense that we aim to define a new type of influence based on the attractiveness model in order to detect those actors who attract new other attractors to participate in the activities of the own community. In addition, our study is related to the approach of [8, 9] in the sense that an actor is influential if he/she is linked from other influential actors. This new type of influence led us to propose a new measure (*HT* measure) to detect those actors, and compare the results with other standard measures. In this paper, we evaluated the performance of our measure in the information diffusion maximization problem by selected sets of top actors based on *HT* measure and other sets which are defined by *T*, Katz measure, PageRank, and other standard measures.

## Approach

The approach of *T* measure provides a new type of influence in online social network in order to emphasize on those actors who attract many outsiders to join the own community in which a specific topic is dealt [2, 3]. Thus, influential actors who are detected by *T* measure are those actors whose tweets spawn many retweets in a way that leads to an increase in the size of social network. *T* measure depends on the decomposition of a topical dataset that is collected from a social network according to the time period of collection.

The basic idea of the dataset decomposition is to analyze a specific event in social media after each slice of time. The aim is to define the actors who affect the size of this event by attracting outsiders to participate. To be more specific, the attractiveness value (*T* value) of the actor *A* in the slice time *t* equals the number of new actors who joined the community in the slice time $t+1$ by establishing new connection with actor *A*.

To formalize our *HT* measure, we will enumerate here briefly some of the concepts that were used to implement *T* measure. The approach of *T* measure is based mainly on the decomposition of a topical dataset that is collected from a social network according to the time period of collection. This time period is referred to by the term *P*-period.

### **Definition 1**

(*P*-*period*) *P*-period is a time duration of the data collection process from social networks.

The definition above is applied to the streaming dataset obtained from online social networks. If we have a historical dataset, *P*-period will be the period between the oldest activity (in Twitter, the activity would be tweet, retweet, reply, etc.) and the newest one in that dataset.

The social networks dataset in this approach is represented by a directed graph which is referred to by *P*-graph.

### **Definition 2**

(*P*-*graph*) *P*-graph is a directed graph constructed from social network data which have been collected during *P*-period. Thus, the collected graph during *P*-period is described by *P*-graph *G*(*V*, *E*), where

- *V* is the set of all actors who joined the community during *P*-period.
- *E* is the set of all connections that have been established between the actors *V* during *P*-period.

Decomposition of a *P*-graph leads to decomposition of the *P*-period into slices of time so that every subgraph is related to a slice. This slice is referred by *P*-slice.

### **Definition 3**

(*P*-*slice*) *P*-slice is a time slice of *P*-period.

If all *P*-slices are equidistant, the *P*-slice is called *EP*-slice.

### **Definition 4**

(*EP*-*slice*) *EP*-slice is a *P*-slice in case all *P*-slices are equidistant.

To ease the definition of subgraphs of this approach, some terms related to actors according to *P*-slices are defined.

### **Definition 5**

(*P*-*actors*) Let $s_1,s_2,\ldots s_n$ be the *P*-slices. For every *i* such that $0 < i \le n$, the *P*-actors $A_i$ is the set of all actors that joined the network until $s_i$.

### **Definition 6**

($P_s$-*actors*) Let $s_1,s_2,\ldots s_n$ be the *P*-slices. For every *i* such that $0 < i \le n$, the $P_s$-actors $A_{s_i}$ is a set of all actors that joined the network between the *P*-slices $s_{i-1}$ and $s_i$.

**Fig. 1:**
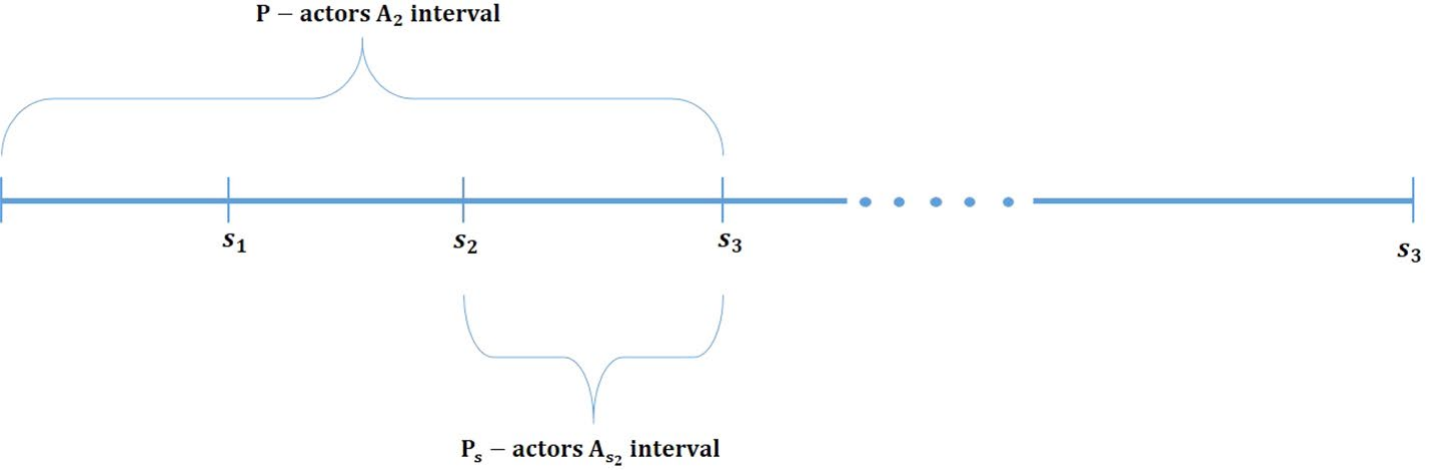
*P*-actors and $P_s$-actors with respect to *P*-slices

Figure 1 shows how the *P*-actors and $P_s$-actors are taken with respect to *P*-slice in this approach. The figure displays the *P*-actors $A_3$ and $P_s$-actors $A_{s_3}$ as an example. $A_3$ is the set of all actors who joined the community until $s_3$ , whereas $A_{s_3}$ joined between *P*-slices $s_2$ and $s_3$.

The subgraphs used in this approach are defined as the following:

### **Definition 7**

(*P*-*subgraph*) *P*-subgraph $G_i(A_i,E_i)$ is a directed subgraph of *P*-graph which is aggregated until *P*-slice $s_i$. Thus, the aggregated subgraph until *P*-slice *i* is described by the *P*-subgraph $G_i(A_i,E_i)$, where

- $A_i$ is the *P*-actors $A_i$.
- $$E_i= \{(a,b) : a,b\in A_i\}$$

### **Definition 8**

(*S*-*subgraph*) The *i*th *S*-subgraph $S_i(A_i,E_{s_i})$ is a subgraph of the *P*-subgraph $G_i(A_i,E_i)$ such that

- $A_i$ is the *P*-actors $A_i$.
- $$E_\mathrm{{si}}= \{(a,b) : a\in A_{i-1} \ {\text{and}} \ b\in A_{s_i}\} \ \cap E_i$$

**Fig. 2:**
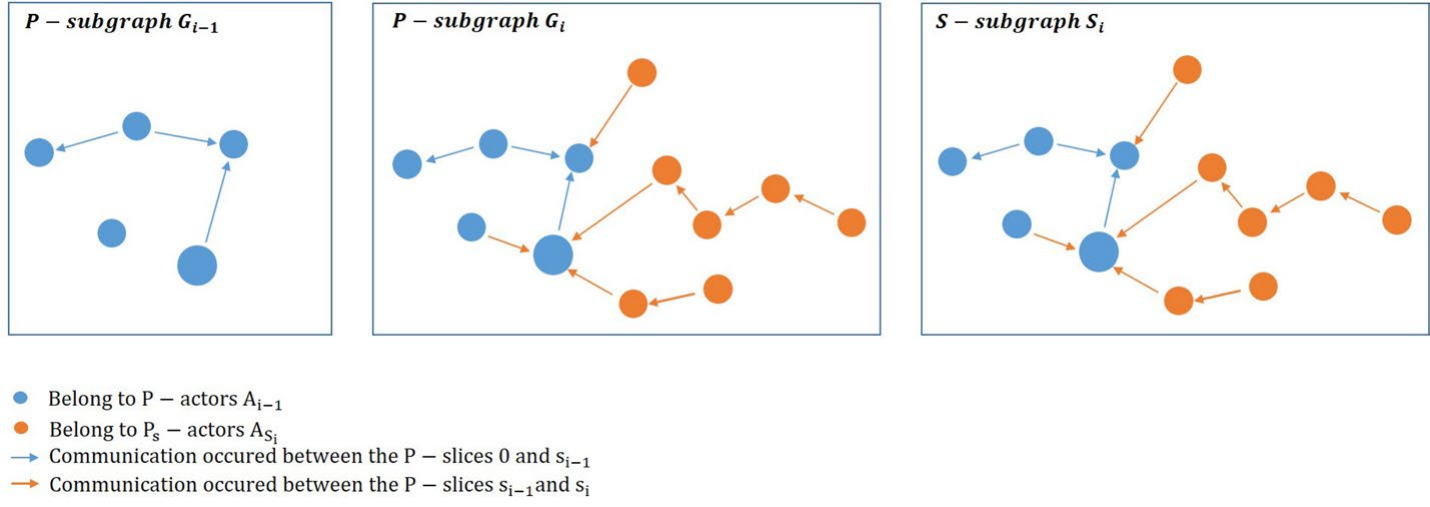
Directed *P*-subgraphs $G_{i-1}$ and $G_{i}$, and directed *S*-subgraph $S_{i}$

Figure 2 shows the difference between *P*-subgraph and *S*-subgraph in this approach, where *n* is the number of *P*-slices and $1<i\le n$. *P*-subgraph $G_{i-1}$ is the *P*-subgraph of the *P*-slice $s_{i-1}$, and *P*-subgraph $G_{i}$ and *S*-subgraph $S_{i}$ are of the *P*-slice $s_{i}$.

Based on the last definitions, we will introduce the implementation of *T* measure and its extended *HT* measure.

**Fig. 3:**
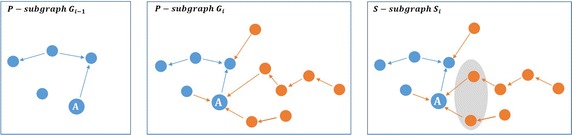
*T* measure evaluation. *T* value of actor *A* is computed from the immediate neighbors who join the network after her/him (located in the *shaded region*)

*T* measure tries to define those actors who attract many actors to the community. Figure 3 shows how the attractiveness value of the actor *A* is calculated with respect to *T* measure.

From Fig. 3, *T* value of the actor *A* in the *P*-subgraph $G_{(i-1)}$ is equal to its indegree value in the* S*—subgraph $S_i$:

1 $$\begin{aligned} T(A_{G_{i-1}})=\text{indegree}\,(A_{S_i}) .\end{aligned}$$

**Fig. 4:**
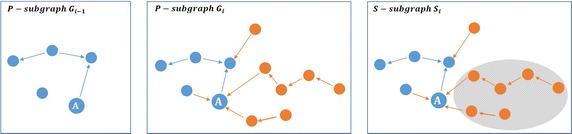
HT measure evaluation. *HT* value of actor *A* is computed from the neighbors of order *n* who join the network after her/him (located in the *shaded region*)

The indegree measure evaluates the number of neighbors of the actor *A* with order 1 (number of the immediate neighbors). In *HT* measure, we will increase the order to include the neighbors with order *m*, where *m* is the maximum neighborhood order. Thus, *HT* measure defines the attractors of attractors. Figure 4 shows the difference between *T* measure and *HT* measure.

From Fig. 4, *HT* value of the actor *A* in the *P*-subgraph $G_{(i-1)}$ is equal to its indegree plus the indegree of his/her neighbors with order *m* in the* S*—subgraph $S_i$.

2 $$\begin{aligned} HT(A_{G_{i-1}})= T(A_{G_{i-1}}) + {\sum \limits _{a\in neighbors(A_{s_i},m)}^{} \text{indegree}\,(a_{S_i})} \end{aligned},$$

where *m* is the maximum neighborhood order.

*HT* and *T* values of the actor *A* in whole *P*-graph *G* are calculated as follows:

3 $$\begin{aligned} T(A_G)={\sum \limits _{i=1}^{n-1} T(A_{G_i})} \end{aligned}$$

4 $$\begin{aligned} HT(A_{G})={\sum \limits _{1}^{n-1} HT(A_{G_i})} \end{aligned},$$

where *n* is the number of slices.

## Evaluation I

In this section, we will describe the evaluation strategy. Furthermore, the experimental results on the datasets will be discussed in this section.

### Evaluation strategy

*HT* measure has been applied to two different datasets.

First, we chose the open source software development project Asterisk. Here, the dataset originated from the communications in the developer mailing lists during 2006 and 2007. The Asterisk dataset contains 13,542 messages and 4694 threads that were discussed by 1324 developers. Two actors are linked if they participated in the same mailing thread. According to our approach and the timestamps in Asterisk dataset, we decomposed the *P*-period into eight *P*-slices. According to Definitions 7 and 8, we got eight *P*-subgraphs and seven *S*-subgraphs.

Second, we gathered a dataset from Twitter via Twitter API from December 31, 2015 to January 06, 2016. This Twitter dataset relates to the hashtag #EndTaizSiege (14,944 actors and 46,552 connections) that comprises a big connected component (containing 84% of actors), singletons (14%), and smaller components (2%). Applying our approach leads to decompose *P*-graph constructed from Twitter dataset into three *P*-subgraphs and two *S*-subgraphs based on three *P*-slices.

**Fig. 5:**
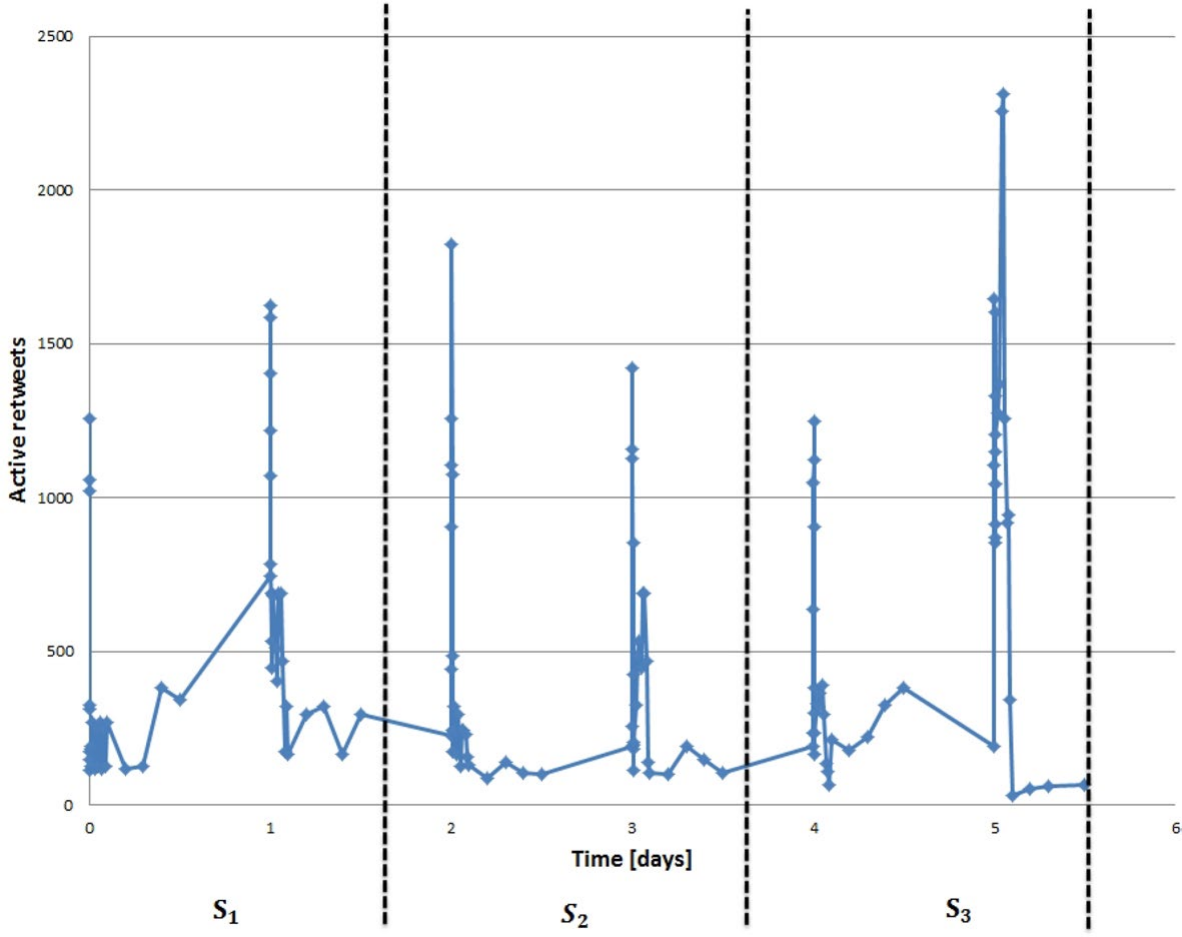
Retweet activities over time in our Twitter dataset

As a matter of fact, the time slicing has been estimated in accordance to the size of dataset using an equal window size for each slice. An example of time slicing manner is described in Fig. 5. Figure 5 shows how the *P*-period with Twitter dataset #EndTaizSiege has been decomposed into equal window size so that we get a fair division of the retweet activities for each time slice.

**Fig. 6:**
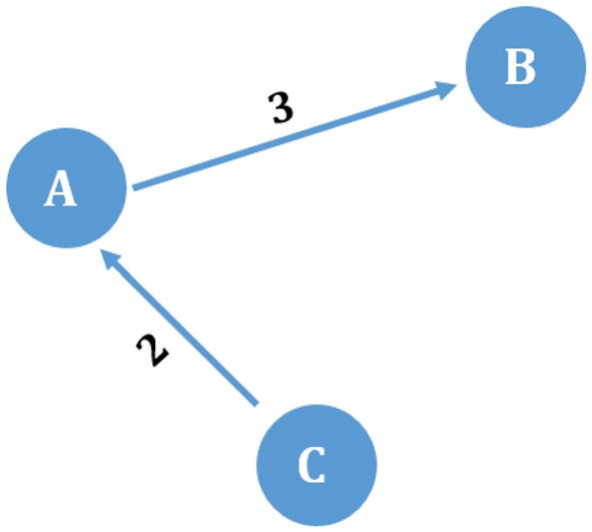
An example of graph representation for our Twitter dataset

The directed weighted *P*-graph of our collected Twitter dataset is constructed based on retweet activities so that actor *A* gets incoming connection from actor *B* if actor *B* retweeted a tweet of actor *A*. The weight of connection refers to the number of retweets between two connected actors. Figure 6 shows an example where actor *A* retweeted 3 tweets of actor *B*, whereas the actor *C* retweeted 2 tweets of the actor *A*.

Boyd et al. [21] argued that retweet relation can be understood as a form of information diffusion and as a means of participating in an event in social media. Thus, we focus on retweet activity as an indicator of attractiveness in the social community.

### Experimental results

We study here the relation between *HT* measure and other influence measures (recursive and other standard measures) in Asterisk and Twitter datasets using Spearman’s rank correlation coefficient $\rho$. The results are shown in Tables 1 and 2.

**Table 1:** Spearman’s rank correlation coefficient over Asterisk dataset

	*HT*	*T*	Degree	Betweenness	Eigenvalue
*HT*	–	0.5807	0.3711	0.4030	0.3479
*T*	–	–	0.643	0.6930	0.574
Degree	–	–	–	0.869	0.910
Betweenness	–	–	–	–	0.716
Eigenvalue	–	–	–	–	–

**Table 2:** Spearman’s rank correlation coefficient over Twitter dataset #EndTaizSiege

	*HT*	*T*	Indegree	Betweenness	Auth	Katz	PageRank
*HT*		0.5024	0.3171	0.2997	0.3249	0.3152	0.3169
*T*			0.5956	0.5401	0.4132	0.6144	0.6114
Indegree				0.598	0.6823	0.9991	0.9973
Betweenness					0.4123	0.6208	0.7508
Auth						0.7569	0.7508
Katz							0.9963
PageRank							

The correlation between *T* measure and other measures was discussed in [2, 3]. According to the correlation values between *HT* measure and other measures, we can notice the following:

- The rank correlation between *HT* and *T* measures is strong ($\rho$ = 0.5). This is reasonable as the *HT* measure is the recursive *T* measure.
- The rank correlation between *HT* and other measures is weak. This leads to the interpretation that our measure is not related to the standard ones. Thus, we can rely on *HT* measure to rank the actors in relation to their attractiveness in a way that is different from standard measures. As a result, these correlation values give us reasonable grounds to use *HT* measure rather than existing measures in the sense that we want to define the attractors in social media networks.

Furthermore, for our Twitter dataset, we applied *HT* measure to verify whether it can detect influential actors. Table 3 shows the description of the top influential actors with respect to *HT*, *T*, Katz centrality, PageRank, indegree, and betweenness measures. The question mark in Table 3 refers to an actor who is not well known as an influential actor within the community. We notice here how the *HT* and *T* measures refer to well-known influential actors within the community, or to the famous news accounts. Unlike other measures, the top ten influential actors with respect to *HT* and *T* measures are well known within the community. In our case, the well-known actors have been recognized based on a local expertise, where they are the most renowned actors in the field of human rights and politics who continually traded their names in the newspapers and news concerning the current situation in Taiz city in Yemen. Their names have not been mentioned explicitly in order to protect their privacy.

**Fig. 7:**
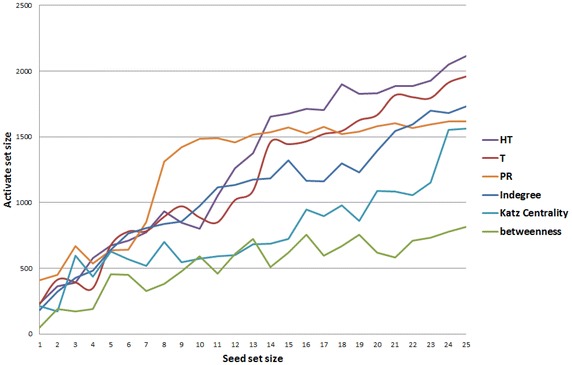
IC model under time-respecting paths with different influence measures over Twitter dataset #EndTaizSiege

**Table 3:** Description of top influential actors according to different influence measures in Twitter dataset #EndTaizSiege

Rank	*HT*	*T*	Indegree	Betweenness	Katz centrality	PageRank
1	News account N1	News account N1	News account N1	?	News account N1	?
2	TV announcer T1	Journalist J1	Journalist J1	?	?	News account N1
3	Journalist J1	TV announcer T1	TV announcer T1	?	Human rights activist H1	TV announcer T1
4	Human rights activist H1	Television reporter R1	Journalist R3	Journalist J2	Journalist J2	Political activist P2
5	Human rights activist H2	Human rights activist H1	Human rights activist H1	?	?	?
6	Television reporter R1	Human rights activist H2	News account N2	?	Television reporter R1	?
7	News account N2	News account N2	Human rights activist H2	Human rights activist H3	Journalist J1	?
8	Journalist J2	Political activist P1	?	TV announcer T1	TV announcer T1	?
9	Political activist P1	Journalist J2	Political activist P1	News account N1	?	News account N3
10	Political activist P2	Political activist P2	?	?	?	Human rights activist H2

## Evaluation II

*T* and *HT* measures are based on a model of actors who attract the outsiders to be active in a community. The idea of information diffusion models is based on the same concept to define the spreaders in a specific community. For this reason, we study our measure in the context of information diffusion to asses how well the *HT* measure is suited to identify influential actors.

We simulate the diffusion of information originating from a seed set of nodes through the Twitter networks using the well-known independent cascade (IC) model [14]. The reason why we use the IC model instead of the LT model is that the linear threshold model is receiver oriented. This means an actor becomes active if a certain fraction of its neighbors are active. This does not account for our purpose where we want to find influential actors who are likely to attract others. The IC model is sender oriented, and thus, is better suited to simulate attraction processes.

In information diffusion, the IC model is proposed where the information flows through cascade over the social network. In IC model, there are two terms which are used to describe the state of the actors. The actor who is influenced by the information is called active, and inactive for the actor who is not influenced. The IC model process starts with activated actors as an initial seed set. In step *s*, an actor *A* will get a single chance to activate each currently inactive neighbor *B*. Actually, the activation process is based on the propagation probability *P* of the actors links. The propagation probability *P* of a link is the probability by which an actor can influence the other actors. In Twitter, we proposed that actor *A* is influenced by actor *B* if he/she retweeted from actor *B* in proportion to the tweets number of actor *B*. So, the propagation probability *P* in IC model is based on our Twitter dataset on the link weight divided by tweets number of target actor.

To compare the performance of actors sets selected by the *HT* measure with other influence measures, we selected sets of top actors based on the *HT*, *T*, PageRank, and Katz centrality measures. As well as, we selected the sets identified by measures that are known to be good heuristics for seed set selection, namely degree and betweenness centrality [22].

### Simulation of attraction processes with time-respecting paths

In this section, we will report results based on simulated attraction processes. To do so, we adapt the IC model that is known to simulate the diffusion of information through a network as described above. Information diffusion and attraction processes have some commonalities but differ on various aspects. In traditional information diffusion models such as the IC model, the network is usually considered as stable in the sense that the set of nodes and the set of edges do not change over time. However, the nodes change their states “inactive” and “active” during the information diffusion process. Attraction, as it is studied in this paper, is similar in the sense that actors who are not part of the community (i.e., do not have contributed a tweet) are inactive while others are considered as active. On the other hand, the original IC model does not account for the fact that the network grows when new actors become attracted to the community. Thus, the IC model was adapted to take into account the creation times of the edges. These time-varying networks have special characteristics regarding reachability of node pairs since a walk on the graph can only take edges with increasing timestamp, which is known as the time-respecting property (see [23]). In this aspect, we added a new activation rule to the IC model which is as follows: the actor who is activated in time *t* cannot activate those actors who have been linked with him/her before the time *t*. To explain this activation rule in more detail, we define the following terms:

#### **Definition 9**

(*Path*-*time*) The path-time of each link in the network is the *P*-slice number in which this link has been created.

#### **Definition 10**

(*Activation*-*time*) The activation-time of each activated actor is the path-time of the link by which this actor has been activated.

Now, we can state that actor *A* cannot activate actor *B* if the link from *B* to *A* has a path-time later than the activation-time of actor *A*.

Using this activation rule, the simulation can be interpreted as an attraction process where actors who are already part of the communities can attract others only if their activity starts after the activator has become active. Algorithm 1 shows the pseudo code of IC model simulator which takes the seed set *S* as a parameter, and returns the number of activated actors by *S*.

The experimental results in the next section support the assumption that the *T* and *HT* measure can identify important attractors in time-varying networks while it boils down to indegree if time is neglected.
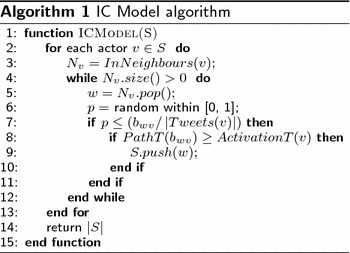


### Experimental results

Here, we considered the dataset of #EndTaizSiege which is related to an organized event in Yemen. Hence, we got a highly connected component that is suitable for the application of our approach which is basically aimed to identify those actors who contribute to attract others to participate in a specific organized event. We simulated the information diffusion based on the IC model with time-respecting paths for seed sets of sizes $n = 1\ldots 25$ which are generated from different influence measures. The diagram in Fig. 7 shows the results of applying IC model on our Twitter dataset with different seed sets which were identified by different influence measures. Comparing with other influence measure, we notice that the *HT* measure yields the best performance in information diffusion under the IC model with time-respecting paths for the seed sizes bigger than 11. Additionally, we statistically verified the results of simulation for each seed set using *T* Test.

- In case of $n > 13$, the differences between *HT* and *T* measures are significant. For example, results for the seed set 12 show that there is a significant difference in the score of* HT* measure (*M* = 1259.95; SD = 291.1128 conditions; *t*(19) = 3.678480757; *P* = 0.000). Table 4 presents the relevant descriptive statistics.
- Furthermore, Table 5 shows that the differences among *HT* and indegree measures are significant in case of $n > 12$.
- As well as, Table 6 shows that the differences among *HT* and PageRank measures are significant in case of $n > 13$.

**Table 4:** *t* test verification for simulation results in case of seed sizes $n\,(n>11)$ among *HT* and *T* measures

Seed_size	*t*	*df*	Sig. (2-tailed)	95% confidence interval		Mean	Mean difference	Std. deviation
				Lower	Upper			
12	3.678480757	19	0.000	1123.705	1396.195	1259.95	239.45	291.1128331
13	4.22734991	19	0.000	1234.088984	1520.11016	1377.4	289.45	306.2106946
14	5.974134667	19	0.000	1585.111672	1717.788328	1651.45	189.35	141.7442007
15	11.96513599	19	0.000	1636.559381	1717.340619	1676.95	230.9	86.30208572
16	12.31058407	19	0.000	1670.410518	1753.889482	1712.15	245.5	89.18418257
17	8.398666846	19	0.000	1657.515863	1746.284137	1701.9	178.1	94.83498133
18	13.79189067	19	0.000	1845.621984	1952.778016	1899.2	353.05	114.4794167
19	13.11261059	19	0.000	1793.720509	1856.179491	1824.95	195.65	66.72762783
20	10.23576478	19	0.000	1797.339971	1863.960029	1830.65	162.9	71.17308406
21	4.625154335	19	0.000	1854.599435	1915.600565	1885.1	67.4	65.17014169
22	5.247863123	19	0.000	1853.214713	1917.985287	1885.6	81.2	69.19720104
23	4.689544215	19	0.000	1840.857049	1911.642951	1876.25	79.3	75.62363526
24	8.32808899	19	0.000	2016.117167	2085.682833	2050.9	138.4	74.3200051
25	9.621110285	19	0.000	2080.48948	2147.21052	2113.85	153.35	71.28096742

**Table 5:** *t* test verification for simulation results in case of seed sizes $n\,(n>12)$ among *HT* and indegree measures

Seed_size	*t*	*df*	Sig. (2-tailed)	95% confidence interval		Mean	Mean difference	Std. deviation
				Lower	Upper			
13	2.9918	19	0.007	1234.088984	1520.711016	1377.4	204.85	306.2106946
14	14.6995	19	0.000	1585.111672	1717.788328	1651.45	465.9	141.7442007
15	19.9816	19	0.000	1636.559381	1717.340619	1676.95	385.6	86.30208572
16	27.3591	19	0.000	1670.410518	1753.889482	1712.15	545.6	89.18418257
17	25.5615	19	0.000	1657.515863	1746.284137	1701.9	542.05	94.83498133
18	23.4663	19	0.000	1845.621984	1952.778016	1899.2	600.7	114.4794167
19	40.0013	19	0.000	1793.720509	1856.179491	1824.95	596.85	66.72762783
20	27.5122	19	0.000	1797.339971	1863.960029	1830.65	437.85	71.17308406
21	23.565	19	0.000	1854.599435	1915.600565	1885.1	343.4	65.17014169
22	18.7068	19	0.000	1853.214713	1917.985287	1885.6	289.45	69.19720104
23	10.5973	19	0.000	1840.857049	1911.642951	1876.25	179.2	75.62363526
24	22.135	19	0.000	2016.117167	2085.682833	2050.9	367.85	74.3200051
25	24.0261	19	0.000	2080.48948	2147.21052	2113.85	382.95	71.28096742

**Table 6:** *T* Test verification for cof seed sizes $n\,(n>12)$ among *HT* and PageRank measures

Seed size	*t*	*df*	Sig. (2-tailed)	95% confidence interval		Mean	Mean difference	Std. deviation
				Lower	Upper			
14	3.6709299	19	0.0001	1585.111672	1717.788328	1651.45	116.35	70.445126
15	5.4229168	19	0.0000	1636.559381	1717.340619	1676.95	104.65	76.684658
16	9.2442207	19	0.0000	1670.410518	1753.889482	1712.15	184.35	53.261618
17	5.8804815	19	0.0000	1657.515863	1746.284137	1701.9	124.7	92.007208
18	13.551641	19	0.0000	1845.621984	1952.778016	1899.2	346.9	98.702157
19	19.100915	19	0.0000	1793.720509	1856.179491	1824.95	285	73.200140
20	15.799773	19	0.0000	1797.339971	1863.960029	1830.65	251.45	72.391189
21	19.255464	19	0.0000	1854.599435	1915.600565	1885.1	280.6	103.614925
22	20.623068	19	0.0000	1853.214713	1917.985287	1885.6	319.1	83.100763
23	16.762312	19	0.0000	1840.857049	1911.642951	1876.25	283.45	84.080538
24	26.106513	19	0.0000	2016.117167	2085.682833	2050.9	433.85	65.184334
25	31.125092	19	0.0000	2080.48948	2147.21052	2113.85	496.1	55.610369

## Conclusions

In summary, we presented in this paper an extended approach to detect influential actors based on the attractiveness model that is introduced with *T* measure. Our approach detects those actors who contribute effectively to increase the size of social network by attracting new attractors to the community in which a specific topic is dealt. Through experiment results we presented how our proposed measure *HT* referred to the influential actors in Twitter dataset. Furthermore, we showed through experiment and statistical tests that the best performance has been yielded by *HT* measure in the influence maximization problem when we took time into account.

Our current work in extending and improving this approach focuses on a differentiation of the role of the actors and different types of communication networks based on the *HT* measure. Furthermore, we plan to study our measure in developing an efficient general strategy for time slicing to determine the time-period decomposition into time slices.
